# Editor's Letter-Box

**Published:** 1894-10-06

**Authors:** 


### EDITOR'S LETTER-BOX.

[Our correspondents are reminded that proxility is a great bar to publication, and that brevity of style and conciseness of statement greatly facilitate early insertion.]

Notice to Correspondent.—M. A. M. D. must give full name and address (not for publication) before his communication can be dealt with. This proceeding is necessary in the case of all communications sent to the Editor.

